# Current Status and Influencing Factors of Snakebite Diagnosis and Treatment Knowledge Among Medical Staff in China: A Cross-Sectional Study

**DOI:** 10.3389/ijph.2023.1606601

**Published:** 2023-12-11

**Authors:** Yanlan Hu, Chuanzhu Lv, Xingyue Song, Yong Gan, Juntao Wang, Wenjie Hao, Lanfen He, Yu Chen, Xiaotong Han, Shijiao Yan

**Affiliations:** ^1^ International School of Public Health and One Health, Hainan Medical University, Haikou, Hainan, China; ^2^ Emergency Medicine Center, Sichuan Provincial People’s Hospital, University of Electronic Science and Technology of China, Chengdu, Sichuan, China; ^3^ Research Unit of Island Emergency Medicine, Chinese Academy of Medical Sciences (No. 2019RU013), Hainan Medical University, Haikou, Hainan, China; ^4^ Key Laboratory of Emergency and Trauma of Ministry of Education, Hainan Medical University, Haikou, Hainan, China; ^5^ Department of Emergency, Hainan Clinical Research Center for Acute and Critical Diseases, The Second Affiliated Hospital of Hainan Medical University, Haikou, Hainan, China; ^6^ Department of Social Medicine and Health Management, School of Public Health, Tongji Medical College, Huazhong University of Science and Technology, Wuhan, Hubei, China; ^7^ Hunan Provincial Key Laboratory of Emergency and Critical Care Metabolomics, Department of Emergency Medicine, Hunan Provincial Institute of Emergency Medicine, Hunan Provincial People’s Hospital, The First Affiliated Hospital, Hunan Normal University, Changsha, Hunan, China

**Keywords:** China, snakebite, medical staff, diagnosis and treatment, envenomation

## Abstract

**Objectives:** This study aimed to determine the current status of the knowledge of diagnosis and treatment of snakebites among medical staff in China and its influencing factors.

**Methods:** A cross-sectional survey of 12,581 medical staff was conducted in 12 provinces in China between June 2022 and February 2023. We analyzed the results using descriptive statistics, T-tests or analysis of variance, and a generalized linear model.

**Results:** The average score of snakebite diagnosis and treatment knowledge among medical staff in China was 3.15 ± 2.15 out of a total score of 12. Through a generalized linear regression model, we found that gender, occupation, region, hospital level, work department, work tenure, training received in the diagnosis and treatment of snakebite, experience in snakebite diagnosis and treatment, availability of antivenom in the unit, and self-evaluation of snakebite treatment ability all affected the medical staff’s scores of snakebite diagnosis and treatment knowledge.

**Conclusion:** The knowledge level of snakebite diagnosis and treatment among Chinese medical staff is generally low, so it is imperative to conduct standardized snakebite diagnosis and treatment training for medical staff.

## Introduction

Snakebites are a severely overlooked problem in subtropical and tropical countries and were reclassified as a neglected tropical disease in 2017 [1]. Snakebites are rarely fatal if treated promptly and effectively. However, in areas with poor health resources, one person dies every 5 minutes from a snakebite and another four are permanently disabled [2]. Rural dwellers, herders, fishermen, and people living in poor housing conditions are at higher risk of snakebite, and as a result the greatest burden in areas with weak health systems and few medical resources [3, 4]. The south of the Yangtze River in China is an area with a high incidence of snakebites, mostly during the busy farming season from April to October, and can occur year-round in tropical and subtropical regions [5], making human-snake interaction common among farmers who mainly engage in agriculture. However, current studies all show that medical staff’s treatment of snakebite patients relies heavily on the patient’s medical history and clinical signs and symptoms [5, 6]. Unfortunately, snake venom detection reagents are not common, which makes it crucial for medical staff to master the knowledge of snakebite diagnosis and treatment, including the venom composition and clinical manifestations of snakebite envenomation. Previous studies have demonstrated the importance of medical staff’s professional knowledge and standardized treatment in improving the outcomes of snakebite patients [7]. A recent global study on the knowledge of medical staff in the diagnosis and treatment of snakebites shows that regardless of the level of development of their respective medical systems, there is a consistent knowledge gap regarding snakebite diagnosis and treatment among medical staff in different countries such as the UK, Cameroon [8], Nigeria [9, 10], Laos [11], Kenya [12], Malawi [13], West African countries [14], Bhutan [15], India [16], etc. Studies have shown that medical staff have obvious deficiencies in the diagnosis and treatment of snakebites. For example, only 23% of medical staff in Bhutan and 48.2% of medical staff in Ghana have adequate knowledge of the diagnosis and treatment of snakebite envenomation. Second, antivenom use is an important part of the knowledge gap. The availability and accessibility of antivenoms are critical to reducing snakebite mortality and disability [17], and lack of knowledge may be one of the reasons for the high mortality rates reported in snakebite epidemiological studies in some countries. For example, 44 cases of snakebite mortality in children were reported in Sri Lanka, with an 11% mortality rate [18], and 2,000 snakebite deaths were reported annually in the Terai district of Nepal [19].

Snakebite is a huge public health crisis. In response to the global snakebite situation, WHO’s Snakebite Envenoming Strategy for Prevention and Control aims to accelerate progress to reduce snakebite-related death and disability by 50% by 2030 [20]. This strategy emphasizes training medical staff in basic snakebite treatment skills, strengthening the healthcare system, increasing regional cooperation, and coordinating medical resources [20, 21]. In this context, medical personnel are important stakeholders in achieving the World Health Organization’s snakebite prevention and control strategy.

At present, there have been no surveys on the status and influencing factors of the diagnosis and treatment of snakebite by medical personnel in China. To achieve the goal of providing safe and effective treatment for snakebite patients, the key is to ensure that the gaps among medical staff in knowledge, training, and patient care quality are reduced. By addressing these knowledge gaps, we can ensure the proper use of antivenoms and improve clinical outcomes for snakebite patients. This study evaluated the *status quo* and influencing factors of snakebite diagnosis and treatment knowledge among medical staff in the south of the Yangtze River, aiming to provide suggestions for the training of snakebite treatment for medical staff in China.

## Methods

### Study Participants and Survey Design

**FIGURE 1:**
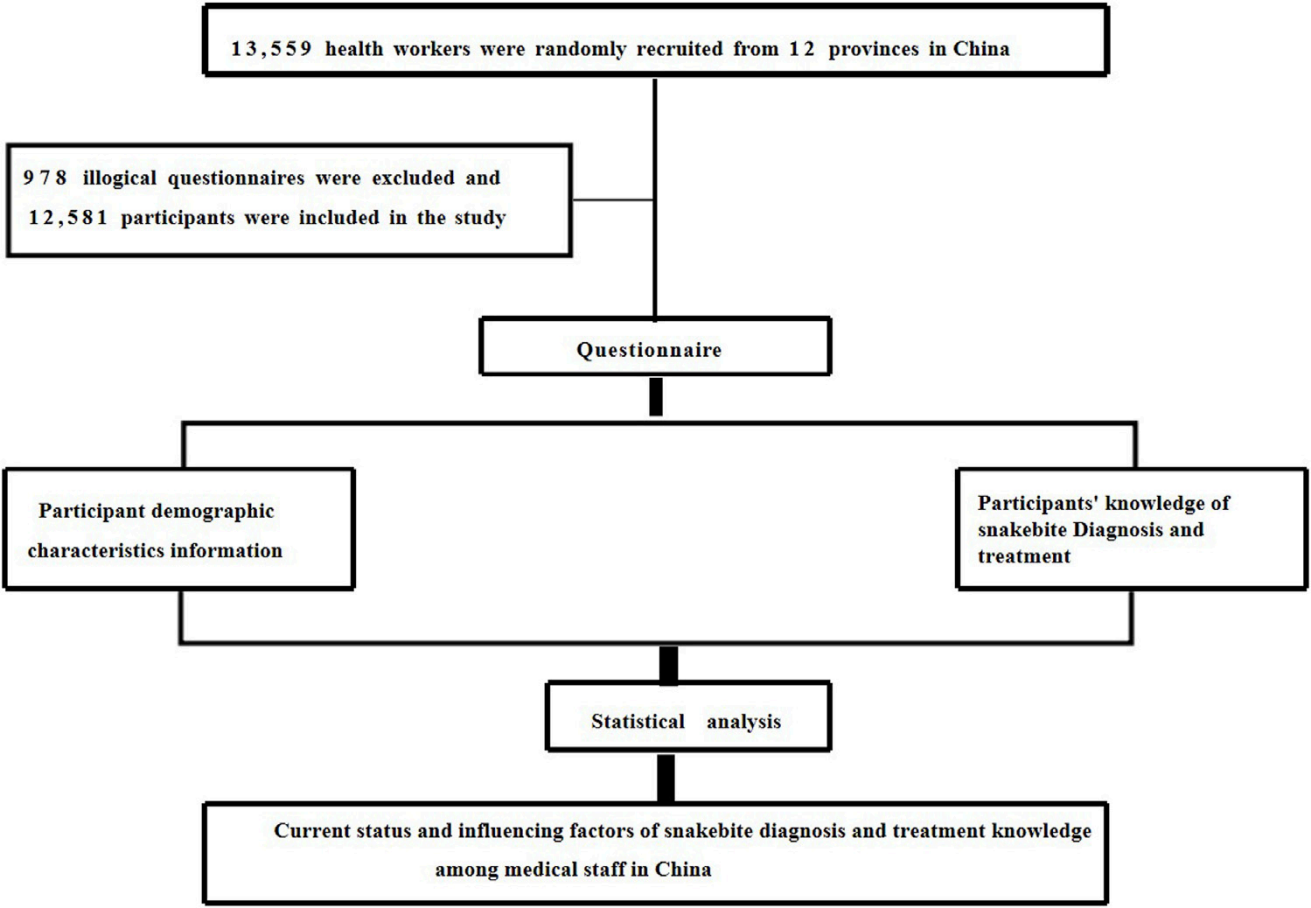
Research process (Haikou, China, 2023).

A cross-sectional study was conducted in China between May 2022 and February 2023, employing a multi-stage random sampling method with complementary snowball sampling to select the research sample. Initially, based on comprehensive literature research and pragmatic considerations, we selected 12 provinces/municipalities/autonomous regions with a notable prevalence of severe snakebite incidents in the Yangtze River Basin and the southern regions. These provinces comprised Hubei, Hunan, Guangdong, Guangxi, Hainan, Zhejiang, Fujian, Jiangxi, Sichuan, Guizhou, Yunnan, and Chongqing. Then, a convenient sampling method was used to select 3 cities within each selected province/municipality/autonomous region, 3 districts/counties within each city, and 3 townships within each district/county. At each stage, 30 to 50 medical staff were randomly selected to participate in the questionnaire survey. Additionally, to supplement the national survey data and cover areas not initially selected, electronic questionnaires were disseminated through popular platforms such as WeChat, employing the snowball sampling method. To prevent the same participant from answering the questionnaire repeatedly, only one questionnaire could be completed per device (such as a smartphone or computer) to prevent duplicate submissions. A total of 13,559 medical workers participated in this survey, 978 questionnaires were excluded due to logical errors, and participants could only submit the questionnaire if they answered all questions. Therefore, there were no missing data in this study. In the end, 12,581 questionnaires were included in this study (the data composition of the 12 provinces is shown in Supplementary Figure S1). The specific process is shown in Figure 1.

### Ethical Statement

This study was approved by the Medical Ethics Committee of Hainan Medical University (HYLL-2022-226) and data collection was approved by the administrative heads of each medical institution. The questionnaire introduction explained the study’s purpose and all individuals participated voluntarily and were anonymous.

### Questionnaire Design and Measurement

The questionnaire was designed based on a literature review, expert consensus on snakebite in China, group discussion, and two rounds of expert letter consultation. The self-assessment was conducted using a five-point Likert scale. In China, all hospitals are divided into three levels, two levels, and one level according to their scale (beds), technical level, management level, equipment conditions, and scientific research capabilities. The knowledge level of snakebite diagnosis and treatment of medical staff was evaluated by questionnaire. In addition, a pilot study was conducted in three hospitals in Hainan Province to improve the quality of the questionnaires. The questionnaire included 1. demographic characteristics of the participants (age, sex, occupation, education level, professional title, region, hospital level, work department, etc.). 2. Work-related information of the participants (work time, whether they had received professional training in snakebites, whether they had experience in diagnosing and treating snakebite patients, whether their unit had antivenom, their evaluation of their current ability to treat snakebites, etc.). 3. 12 questions about snakebite diagnosis and treatment, including knowledge of snake species classification, symptom identification, principles of serum use, and principles of snakebite treatment. 4. Education and training content, first-aid attitudes and behaviors (first aid theoretical knowledge mastery, willingness to train in first aid knowledge, single first aid operational skills mastery, single first aid operational skills training needs). 5. working status and 6. a job satisfaction survey. The demographic characteristics, work-related information, and information on the snakebite expertise of participants were selected for analysis in this study.

### Statistical Analysis

In this study, the Social Science Statistical Package (SPSS) version 22 was used to conduct a descriptive analysis of the demographic characteristics and job-related information of medical staff, to understand the baseline distribution of relevant information of medical staff participating in this survey. Knowledge of snakebite diagnosis and treatment was analyzed by calculating scores from 12 diagnosis and treatment questions. Incorrect answers were assigned a score of 0 and correct answers were assigned a score of 1 (all correct items were identified in the case of multiple core correct answers, otherwise the score was 0), and an overall personal knowledge score was calculated for all respondents, with a maximum possible score of 12 and a minimum possible score of 0). Each question was equally important. Continuous variables were expressed as mean and standard deviation and categorical variables were expressed as frequency and percentage. T-tests or analysis of variance (ANOVA) were used to compare the scores of snakebite diagnosis and treatment knowledge of medical staff among different groups. When the homogeneity test of variance was significant, separate Welch tests were used. To test whether there was collinearity between the variables, the multicollinearity of the independent variables was tested by calculating the variance inflation factor (VIF). Because the dependent variable is the non-normal distribution of snakebite diagnosis and treatment knowledge score, the generalized linear regression model was used to test the relationship between the independent variables and the score of snakebite diagnosis and treatment knowledge. All statistics were two-tailed, and a *p*-value <0.05 was considered statistically significant.

## Results

### Sociodemographic Characteristics and Scores of the Participants

As shown in Table 1, a total of 12,581 medical personnel participated in the survey, the majority of which (7,849, 62.39%) were female, of whom 8,295 (65.93%) had a bachelor’s degree or above. 2,635 (20.95%) medical staff self-rated their ability to treat snakebite as proficient. Of these, 5,147 (40.91%) participants were from the emergency department, only 5,412 (43.02%) medical staff had received training in snakebite diagnosis and treatment, and 6,128 (48.71%) medical staff had antivenoms in their workplace. The average score of snakebite diagnosis and treatment knowledge of Chinese medical staff was Mean ± SD, 3.15 ± 2.15) among which the maximum score was 12 and the minimum score was 0 (The scores of snakebite diagnosis and treatment knowledge of medical staff in 12 provinces are shown in Supplementary Table S3). The score of doctors’ knowledge of snakebite diagnosis and treatment was Mean ± D, 3.53 ± 2.28, Emergency department medical staff diagnosis and treatment knowledge scored Mean ± SD, 3.5 ± 2.30, and medical staff who had received training in the diagnosis and treatment of snakebite scored Mean ± SD, 3.77 ± 2.11. Univariate analysis results showed that the score of snakebite diagnosis and treatment knowledge was related to working time, region, gender, occupation, education level, working department, hospital grade, professional title, participation in the training of snakebite diagnosis and treatment knowledge, experience in snakebite diagnosis and treatment, equipment of work unit with antivenom, and self-evaluation of snakebite treatment ability (*p* < 0. 05). The accuracy rate of the principle of snakebite treatment was 8,435 (67%), and the accuracy rate of the method of antivenom usage was 7,154 (56.9%). The accuracy rate of snakebite wound shape was 166 (1.3%), and the accuracy rate of blood-type venomous snakebite symptom recognition was 1,314 (10.4%). The accuracy rates of specific questions and answers on the knowledge of snakebite diagnosis and treatment are shown in Supplementary Table S1.

**TABLE 1:** Sociodemographic characteristics and snakebite diagnosis and treatment knowledge scores of the participants. China, May 2022 and February 2023.

Variable	N	Percentage	Scores (*M ± SD*)	Minimum value	Maximum values	Statistical value(*F*/*t*)	*p*-value
Total	12,581	100	3.15 ± 2.15	0	12		
Age						1.37	0.253
18–29	4,204	33.42	3.11 ± 2.14	0	10		
30–44	6,286	49.96	3.18 ± 2.18	0	12		
Over 44	2,091	16.62	3.14 ± 2.14	0	12		
Work tenure (years)						109.38	<0.001
<10	6,586	52.35	2.89 ± 2.14	0	11		
10∼19	3,585	28.5	3.33 ± 2.15	0	12		
≥20	2,410	19.15	3.57 ± 2.10	0	12		
Region						28.95	<0.001
East China	2,597	20.64	2.90 ± 2.38	0	11		
Central China	4,821	38.32	3.11 ± 2.05	0	12		
Western China	5,163	41.04	3.3 ± 2.12	0	12		
Gender						8.79	<0.001
Male	4,732	37.61	3.37 ± 2.34	0	12		
Female	7,849	62.39	3.01 ± 2.02	0	12		
Occupation						184.308	<0.001
Doctor	5,782	45.96	3.53 ± 2.28	0	12		
Nurse	6,027	47.9	2.86 ± 1.99	0	10		
Other medical profession	772	6.14	2.50 ± 1.94	0	10		
Education level						162.17	<0.001
Lower than bachelor	4,286	34.07	2.69 ± 1.98	0	10		
Bachelor’s degree	7,455	59.26	3.38 ± 2.14	0	12		
Master’s degree or higher	840	6.67	3.42 ± 2.74	0	11		
Level of hospital						75.24	<0.001
Tertiary hospital	5,112	40.63	3.42 ± 2.33	0	12		
Secondary hospital	4,884	38.82	3.01 ± 2.05	0	12		
Primary hospital	2,585	20.55	2.85 ± 1.93	0	10		
Work department						120.88	<0.001
Emergency department	5,147	40.91	3.50 ± 2.30	0	12		
General surgery	1,117	8.88	2.41 ± 2.33	0	12		
General practice	2004	15.93	2.64 ± 1.99	0	10		
Other departments	4,313	34.28	3.15 ± 1.91	0	10		
Professional title						99.16	<0.001
Junior less	1,910	15.18	2.49 ± 2.02	0	10		
Junior	5,467	43.45	3.09 ± 2.03	0	10		
Intermediate	3,626	28.82	3.36 ± 2.18	0	12		
Senior	1,578	12.55	3.64 ± 2.46	0	12		
Training in the diagnosis and treatment of snakebites						29.13	<0.001
Yes	5,412	43.02	3.77 ± 2.11	0	12		
No	7,168	56.98	3.67 ± 2.07	0	10		
Experiences in treating patients with snakebites						29.04	<0.001
Yes	4,679	37.19	3.85 ± 2.23	0	12		
No	7,902	62.81	2.73 ± 2.00	0	10		
The hospital where they work has antivenom						667.14	<0.001
Yes	6,128	48.71	3.76 ± 2.12	0	12		
No	4,352	34.59	2.83 ± 2.00	0	10		
Not clear	2,101	16.7	2.01 ± 1.95	0	9		
Know the way to obtain antivenom							
Yes	6,368	50.62	3.17 ± 2.15	0	12	1.39	0.166
No	6,213	49.38	3.12 ± 2.17	0	10		
Self-evaluation of snakebite treatment ability						226.70	<0.001
Very skilled	683	5.43	3.88 ± 2.71	0	12		
Proficient	1,952	15.52	4.02 ± 2.27	0	11		
Moderately skilled	4,570	36.32	3.26 ± 2.01	0	10		
Unskilled	3,933	31.26	2.87 ± 1.98	0	10		
Very unskilled	1,443	11.47	2.01 ± 1.96	0	9		

Medical staff snakebite diagnosis and treatment knowledge scores from 12 questions: Incorrect answers were assigned a score of 0 and correct answers were assigned a score of 1 (all correct items were identified in the case of multiple core correct answers, otherwise the score was 0. *M*, Mean value, *SD*, Standard deviation. Maximum value, the maximum score of medical staff’s knowledge of snakebite diagnosis and treatment, Minimum value, the minimum score of medical staff’s knowledge of snakebite diagnosis and treatment.

### Results of Generalized Linear Regression Analysis

The results of the multicollinearity test showed a maximum VIF = 1.68 and minimum VIF = 1.14 (see Supplementary Table S2), and the generalized linear model analysis results are shown in Table 2. In terms of individual factors, male medical staff (*p* < 0.001, β = −0. 23) scored lower in the knowledge of snakebite diagnosis and treatment compared to female medical staff; compared to other medical professionals, doctors (*p* < 0.001, β = 0.76) scored higher knowledge of snakebites diagnosis and treatment; compared to those with a master’s degree or higher, those with a lower than Bachelor (*p* = 0. 001, β = −012) scored lower; compared with tertiary hospitals, medical staff in primary hospitals (*p* < 0. 001, β = 0.22) scored higher in the diagnosis and treatment of snakebite; compared to other departments, and emergency (*p* < 0.001, β = −0.25), general surgery (*p* < 0.001, β = −0.87), and general practice (*p* < 0.001, β = −0.54) medical staff scored lower knowledge of snakebites diagnosis and treatment. Compared with the western region, the score of snakebite diagnosis and treatment knowledge of medical staff in the eastern region (*p* < 0.001, β = −0.44) was lower. In terms of work-related information, medical staff with less than 10 years of service (*p* < 0.001, β = −0.18) scored lower on snakebite diagnosis and treatment knowledge than medical staff with 20 years or more of service; in medical staff who had received training in the diagnosis and treatment of snakebite (*p* < 0.001, β = 0.53), and those who had experience in the diagnosis and treatment of snakebites (*p* < 0.001, β = 0.34), the score of snakebite diagnosis and treatment knowledge was higher. Compared with those who did not know whether the unit was equipped with snake venom, the medical staff who were equipped with antivenom in the work unit (*p* < 0.001, β = 1.02) scored higher on the knowledge of snakebite diagnosis and treatment. Compared with medical staff who were not skilled in the self-treatment ability evaluation, those who declared self-rated proficiency (*p* < 0.001, β = 0.89) had higher scores in snakebite diagnosis and treatment knowledge.

**TABLE 2:** Correlation between independent predictor variables and score of snakebite diagnosis and treatment knowledge. China, May 2022 and February 2023.

Variables	B	SE	Wald	*p-*value
Work tenure (year) (Ref: ≥20)				
<10	−0.18	0.06	58.43	<0.01
10∼19	0.26	0.06	22.15	<0.01
Region (Ref: Western China)				
Eastern China	−0.44	0.05	55.29	<0.01
Central China	−0.26	0.04	11.48	0.01
Gender (Ref: Female)				
Male	−0.23	0.05	23.89	<0.01
Occupations (Ref: Others profession)				
Doctor	0.76	0.08	87.94	<0.01
Nurse	0.11	0.08	1.75	0.19
Education level (Ref: Master degree or higher)				
Lower than bachelor	−0.12	0.08	2.01	0.15
Bachelor degree	0.13	0.08	2.77	0.1
Level of hospital (Ref: Tertiary hospital)				
Primary hospital	0.22	0.06	12.39	<0.01
Second Hospital	−0.05	0.04	1.32	0.25
Work department (Ref: Other Departments)				
Emergency Department	−0.25	0.05	23.41	<0.01
General surgery	−0.87	0.07	160.43	<0.01
General practice	−0.54	0.06	84.73	<0.01
Professional title (Ref: Senior)				
Junior less	−0.20	0.08	5.99	0.01
Junior	0.04	0.07	0.30	0.59
Intermediate	0.05	0.06	0.54	0.46
Training in the diagnosis and treatment of snakebites (Ref: No)				
Yes	0.53	0.04	165.52	<0.01
Experiences of treating patients with snakebite (Ref: NO)				
Yes	0.34	0.05	53.20	<0.01
The hospital where they work has antivenom (Ref: Not clear)				
Yes	1.02	0.06	332.47	<0.01
No	0.45	0.06	66.63	<0.01
Evaluation of their current ability to treat snakebites (Ref: Very unskilled)				
Very skilled	0.68	0.10	44.75	<0.01
Proficiency	0.89	0.08	124.72	<0.01
General	0.61	0.06	92.30	<0.01
Unskilled	0.56	0.06	85.96	<0.01

Ref, Reference B, Regression coefficient SE, Standard error.

## Discussion

This study evaluated the *status quo* of snakebite diagnosis and treatment knowledge of medical staff in 12 provinces in China and related factors. The results showed that the mean score of snakebite diagnosis and treatment knowledge among medical staff in China was 3.15 ± 2.15 out of a total score of 12. The survey also found that the accuracy rate of medical staff in the treatment of snakebites was 8,435 (67%), and the accuracy rate of the use of antivenom was 7,154 (56.9%). However, the recognition accuracy rate of snakebite wound shape was 166 (1.3%), and the recognition accuracy rate of blood-type venomous snakebite symptoms was 1,314 (10.4%). The identification of various types of snakebite symptoms by Chinese medical staff needs to be further strengthened. In particular, the current antivenom in China is a monovalent antivenom, which requires medical staff to select the appropriate anti-venom serum according to the patient’s symptom identification. According to this survey, only 5,412 (43.02%) medical staff had received training in the diagnosis and treatment of snakebite, and the score of snakebite diagnosis and treatment knowledge of medical staff who had received training was only 3.77 ± 2.11. Snakebite is a critical emergency, and medical staff’s knowledge of diagnosis and treatment is a guarantee of the provision of high-quality medical services [22]. It is well-established that access to high-quality healthcare services can improve snakebite survival rates, reduce disability, and decrease sequelae [23]. This shows that China currently needs to train medical staff in the diagnosis and treatment of snakebites. A study in Ghana comparing outcomes before and after training medical staff on snakebite diagnosis and treatment found that snakebite complications and mortality decreased after training [9]. However, while carrying out training, we also need to pay attention to the quality of training and effectively strengthen the ability of medical staff to diagnose and treat snakebites.

Our study found that the score of snakebite knowledge of medical staff in the eastern region was 0.44 lower than that of the western region, and the score of snakebite knowledge of medical staff in the central region was 0.26 lower. Among them, the average score of snakebite diagnosis and treatment knowledge of medical staff in Zhejiang Province and Fujian Province in the eastern region was the lowest, and the score of snakebite knowledge of medical staff in Sichuan Province was the highest in the western region. The difference in the knowledge level of snakebite diagnosis and treatment among medical staff in different regions may be that the western and central regions are mountainous [24], the level of economic development is lower than that of the eastern region, and there are more residents engaged in agricultural work than in the eastern region. The epidemiological data on snake bites in China show that agricultural workers are at high risk of snakebites [25, 26], so the number of snakebite patients in the western region is also higher than that in the eastern region [27]. The number of snakebite patients received by medical staff may affect the knowledge level of snakebite diagnosis and treatment of medical staff.

Snakebite is a serious emergency and when patients are snakebitten, the first place of treatment for these high-risk groups should be the primary medical unit or the emergency department. Antivenom is the only specific treatment method that can be used for snakebites [28]. For patients with snakebites, the availability and accessibility of safe and effective antivenom are also key elements for the treatment of snakebites [29]. Our survey found that 7,434 (59.09%) snakebites were treated in other departments, and 6,453 (51.29%) medical units were not equipped with antivenom. In the multivariate analysis, we found that compared with other snakebite treatment departments, the score of snakebite diagnosis and treatment knowledge of medical staff in the emergency department was 0.25 lower. Compared with the medical staff without antivenom in the work unit, the score of snakebite diagnosis and treatment knowledge of medical staff with antivenom in the work unit was 1.02 higher. The level of medical institutions, different departments, and work units equipped with antivenom affects the scores of medical staff in the diagnosis and treatment of snakebite, which may be related to the following reasons. First of all, there is still a phenomenon of unbalanced distribution of medical resources due to unbalanced regional economic development in China [30], especially the allocation of antivenom resources [31]. For medical staff, units equipped with antivenom will encourage medical staff to actively pay attention to the knowledge of snakebite diagnosis and treatment, and will also affect their knowledge score of snakebite diagnosis and treatment. For patients with snakebites, rapid access to effective treatment is essential. Delayed treatment may increase the impact of venom on patients [32], but China’s health decision-making departments do not require emergency departments and primary medical emergency centers to configure matching first-aid drugs. Second, China’s medical insurance department did not include snakebites in emergency-related diseases for medical insurance reimbursement, resulting in different departments receiving snakebite patients. Finally, the high-risk groups of snakebite are preferred to primary medical institutions for treatment after snakebite. Medical staff in primary medical institutions have more opportunities to contact snakebite patients. However, because most snakebite patients do not regard snakebite as an emergency event, pre-hospital delays cause symptoms to increase and patients need to be referred to higher-level hospitals for treatment [33, 34], which may lead to differences in the knowledge of snakebite diagnosis and treatment among medical staff at different levels. Our findings are consistent with those reported in Nigeria [10]. As a country at risk of snakebite, China’s current snakebite diagnosis and treatment capabilities of medical staff and the configuration of antivenom need to be further improved.

Our study found that more than 9,946 (79.05%) medical staff lacked confidence in self-assessment of snakebite diagnosis and treatment knowledge. Previous studies have shown that a lack of confidence among medical staff may increase the incidence of medical errors and put patients at risk [35, 36]. A study in Nepal reported that the availability of data affects the level of medical staff’s knowledge of snakebite diagnosis and treatment [37]. The absence of guidelines and teaching materials specific to snakebite diagnosis and treatment in China may be contributing to this lack of knowledge among medical staff. To address this, it is crucial to provide targeted training and instructional materials to improve the medical staff’s understanding and confidence in snakebite treatment.

Snakebite is a potential occupational hazard. With the development of agriculture, livestock, and fisheries in our country, the conflict between humans and snakes will continue. The study identified several factors that influenced the knowledge of snakebite diagnosis and treatment among medical staff, including working years, region, gender, occupation, education level, working department, working hospital level, professional title, participation in snakebite training, experience in snakebite diagnosis and treatment, availability of antivenom at the workplace, and self-evaluation of snakebite diagnosis and treatment ability. This information can be used as a reference for future training to improve the diagnosis and treatment of snakebite. Among them, the score of doctors’ knowledge of snakebite diagnosis and treatment is higher than that of other medical workers, which may be because doctors play a leading role in the treatment of snakebites [9], which is consistent with previous research results. A sound health system is the basis for improving snakebite diagnosis and treatment capacity, which means strengthening the entire health system, especially the capacity of doctors. Unlike some neglected tropical diseases, snakebite is impossible to eliminate. Venomous snakes play an important role in complex ecosystems, including natural biological control of agricultural pests (such as rodents), yet snakebite can be effectively controlled through innovative and improved disease management, research into diagnosis, treatment, and control, and improved availability, accessibility, and affordability of antivenom [38], as well as by reducing the physical, psychological, and socio-economic burden of snakebite. Therefore, based on the current status of snakebite diagnosis and treatment among medical staff in China and the allocation of anti-venom serum, how to meet the medical needs of people at high risk of snakebite envenomation, how to urge the relevant departments of the state to develop uniform training materials, how to carry out standardized training on the diagnosis and treatment of snakebite envenomation for medical staff, and how to enable different social groups to obtain the necessary medical and health services on a relatively reasonable basis are urgent problems in China. These issues require urgent attention from relevant health authorities in China, with increased policy support, to achieve the vision of zero deaths and disabilities for snakebite patients.

### Strengths and Limitations

The strength of this study lies in its novelty as the first report on the current status of snakebite diagnosis and treatment knowledge among medical staff in China and its associated factors. In addition, the participants were distributed across multiple provinces and cities, and the large sample size could enhance the statistical power of the snakebite diagnosis and treatment knowledge of the medical staff. Therefore, the results of this study are representative. However, our study also has some limitations. First, as the survey was anonymous, it was impossible to verify the authenticity of the data provided by the respondents. Second, the study relies on self-reported data through questionnaires, making it challenging to objectively evaluate the level of snakebite management training received by each respondent. Lastly, because there is currently no unified snakebite treatment program in China, the specific topics of our snakebite expertise involve more knowledge of diagnosis.

### Conclusion

The average score of snakebite diagnosis and treatment knowledge among medical staff in China was 3.15 ± 2.15 out of a total score of 12. We found that gender, occupation, region, hospital level, work department, work tenure, training received in the diagnosis and treatment of snakebite, experience in snakebite diagnosis and treatment, availability of antivenom in the unit, and self-evaluation of snakebite treatment ability all affected medical staff’s scores regarding snakebite diagnosis and treatment knowledge. In order to further improve the knowledge of Chinese medical staff on the diagnosis and treatment of snakebites, it is necessary to carry out training in the diagnosis and treatment of snakebites, especially the identification of symptoms of different types of snakebites.
