# Supplementary material for: Current Status and Influencing Factors of Snakebite Diagnosis and Treatment Knowledge Among Medical Staff in China: A Cross-Sectional Study
Source: Int J Public Health. 2023 Dec 11;68:1606601. doi: 10.3389/ijph.2023.1606601 (PMC10749458; doi:10.3389/ijph.2023.1606601)
Supplement: Supplementary file 1 [file Table3.DOC]

**Supplementary Table3. Scores of snakebite diagnosis and treatment knowledge of medical staff in 12 provinces**

| **Province** | **N** | **Scores( M ± SD)** | **minimum value** | **maximum values** |
| --- | --- | --- | --- | --- |
| Hubei | 3239 | 3.18 ± 1.99 | 0 | 11 |
| Sichuan | 1972 | 3.65 ± 1.97 | 0 | 12 |
| Hunan | 1340 | 2.80 ± 2.17 | 0 | 10 |
| Guangdong | 1218 | 3.51 ± 2.66 | 0 | 11 |
| Guizhou | 1013 | 3.38 ± 1.84 | 0 | 11 |
| Yunnan | 921 | 3.26 ± 1.72 | 0 | 10 |
| Guangxi | 864 | 3.34 ± 2.11 | 0 | 10 |
| Zhejiang | 640 | 2.02 ± 2.36 | 0 | 10 |
| Fujain | 630 | 2.07 ± 2.39 | 0 | 10 |
| Chongqing | 393 | 2.91±1.92 | 0 | 10 |
| Jaingxi | 242 | 3.06 ± 2.36 | 0 | 12 |
| Hainan | 109 | 2.53 ± 2.19 | 0 | 8 |

Scores, Medical staff snakebite diagnosis and treatment knowledge score, that is 12 diagnosis and treatment questions, the wrong answers ween a score of 0, the correct answers were given a score of 1 (all correct items were identified in the case of multiple core correct answers, otherwise the score was 0) *M*, Mean value *SD*,Standard deviation Maximumvalue, The maximum score of medical staff 's knowledge of snakebite diagnosis and treatment Minimum value, The minimum score of medical staff 's knowledge of snakebite diagnosis and treatment
