# Supplementary figures and images for: Current Status and Influencing Factors of Snakebite Diagnosis and Treatment Knowledge Among Medical Staff in China: A Cross-Sectional Study
Source: Int J Public Health. 2023 Dec 11;68:1606601. doi: 10.3389/ijph.2023.1606601 (PMC10749458; doi:10.3389/ijph.2023.1606601)

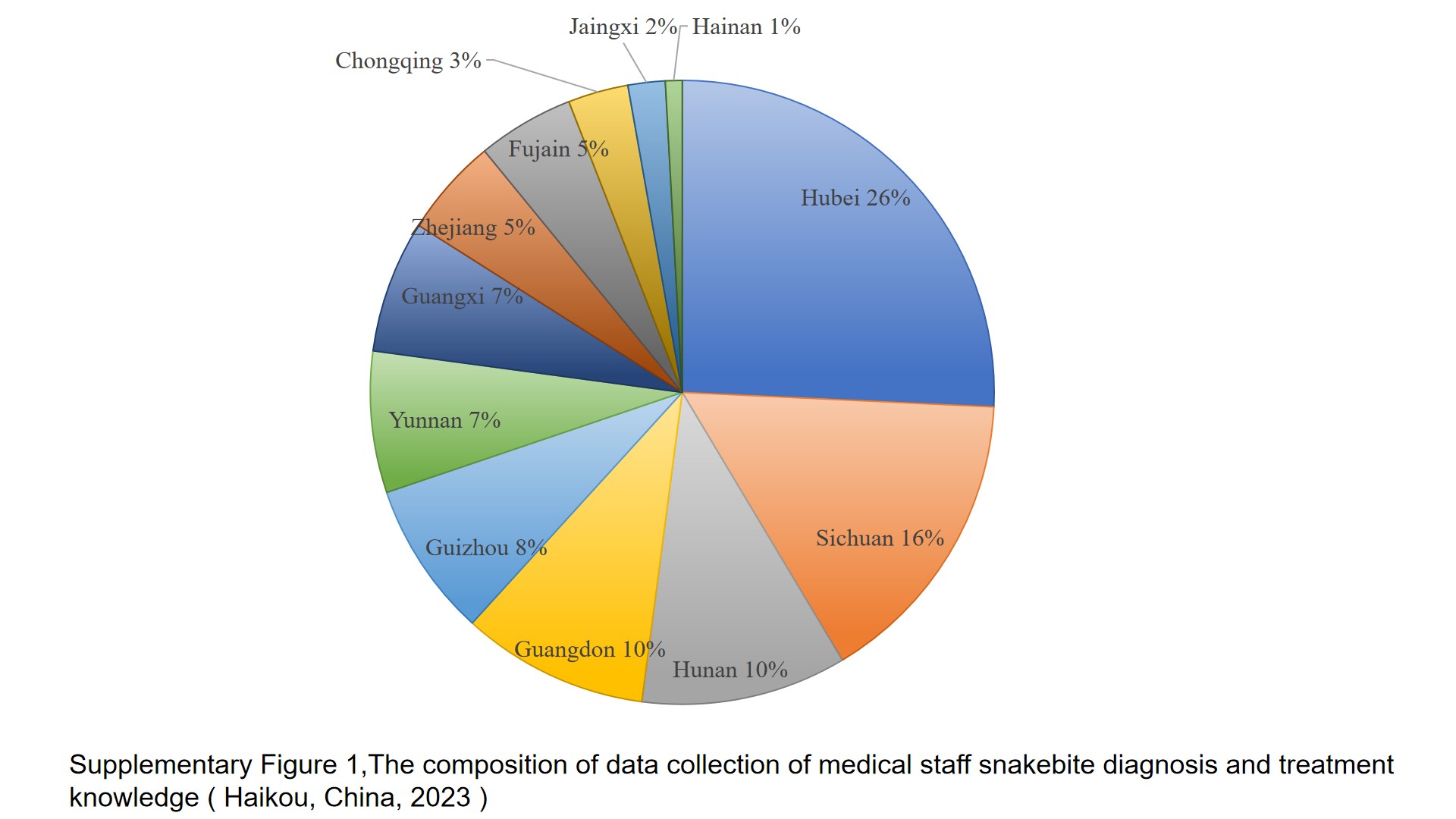

Supplement: Supplementary file 2 [file Image1.JPEG]
