# Supplementary material for: Current Status and Influencing Factors of Snakebite Diagnosis and Treatment Knowledge Among Medical Staff in China: A Cross-Sectional Study
Source: Int J Public Health. 2023 Dec 11;68:1606601. doi: 10.3389/ijph.2023.1606601 (PMC10749458; doi:10.3389/ijph.2023.1606601)
Supplement: Supplementary file 3 [file Table1.DOC]

| **Supplementary Table1.Snakebite diagnosis and treatment knowledge questions and proportions of correct and incorrect answers** | | | |  |
| --- | --- | --- | --- | --- |
| **Question** | **Correct answer**  **N(%)** | **Incorrect answer**  **N(%)** |  |  |
| Venomous snake classification | 7865（62.5） | 4716（37.5） |  |  |
| What are the common Neurotoxicen venoming snakes | 1566（12.4） | 11015（87.6） |  |  |
| When will systemic envenoming symptoms occur after being bitten by a neurotoxic snake | 2899（23.0） | 9682（77.0） |  |  |
| What are the common Haemotoxic venoming snakes | 774（6.2） | 11807（93.8） |  |  |
| What are the common cytotoxic venomous snakes | 1520（12.1） | 11061（87.9） |  |  |
| What are the common mixed venomous snakes | 468（3.7） | 12113（96.3） |  |  |
| What are the general symptoms of haemotoxic venoming snakebite | 1314（10.4） | 11267（89.6） |  |  |
| What are the general symptoms after Neurotoxicen venoming snakebite | 2122（16.9） | 10459（83.1） |  |  |
| What are the general symptoms of Cytotoxic venoming snakebite | 5300（42.1） | 7281（57.9） |  |  |
| Snakebite wound shape | 166（1.3） | 12415（98.7） |  |  |
| The principle of treatment of venomous snakebites | 8435（67.0） | 4146（33.0） |  |  |
| Indications for use of antivenom | 7154（56.9） | 5427（43.1） |  |  |

| Supplementary Table 2. Multicollinearity test results | | |
| --- | --- | --- |
| **Variable** | **Tolerance** | **Variance**  **inflation factor** |
|
| Work experience | 0.67 | 1.50 |
| Region | 0.88 | 1.14 |
| Sex | 0.73 | 1.37 |
| Occupation | 0.66 | 1.53 |
| Education level | 0.72 | 1.38 |
| Hospital level | 0.63 | 1.59 |
| Medicine department | 0.68 | 1.48 |
| Professional title | 0.60 | 1.68 |
| Trained in snakebite expertise | 0.74 | 1.36 |
| Experiences in treating patients with snakebites | 0.60 | 1.67 |
| Availability of antivenom at the hospital | 0.74 | 1.36 |
| Evaluation of their current ability to treat snakebites | 0.60 | 1.67 |
