# Supplementary material for: Current Status and Influencing Factors of Snakebite Diagnosis and Treatment Knowledge Among Medical Staff in China: A Cross-Sectional Study
Source: Int J Public Health. 2023 Dec 11;68:1606601. doi: 10.3389/ijph.2023.1606601 (PMC10749458; doi:10.3389/ijph.2023.1606601)
Supplement: Supplementary file 4 [file DataSheet1.pdf]

Supplementary File 1. Questionnaire

Questionnaire on diagnosis and treatment knowledge of snakebite among medical staff

Dear colleagues in medical institutions,
Hello! Snakebite is a neglected tropical disease, and the knowledge of snakebite diagnosis and treatment by medical staff is crucial to improve the prognosis of patients. In order to understand the current situation of the knowledge of snakebite diagnosis and treatment by medical staff, the establishment and improvement of continuing education system for medical staff in medical institutions is an important part of the in-depth reform of medical reform in China. Based on this, the Emergency Medicine Branch of the Chinese Medical Association has formulated the following questionnaire to invite your participation. Your views are vital to the development of continuing education content on snakebite for medical staff! Please mark "√" on the corresponding alternative project number according to your personal experience. We use an anonymous form to ensure the confidentiality of the information you fill in. Thank you for your understanding and support!
Emergency Medicine Branch of Chinese Medical Association

Background

1. Age : ___ years. (Please fill in the whole number)
2. The number of years you have been engaged in medical and health work :________ years. (Please fill in the whole number, more than half a year please fill in 1 year)
3. The location of the medical unit where you currently work :________ province _________ city _________ county/district.
4. Your gender is?
   ○ Male
   ○ Female
5. Your occupation type is?
   ○ Doctor
   ○ Nurse
   ○ Other (please specify) _________
6. Your highest degree is?

- ○ Technical Secondary School Degree
- ○ Junior College
- ○ Undergraduate Degree
- ○ Master's Degree
- ○ Doctor Degree

7. The level of your hospital?
   - ○ Class A tertiary hospital
   - ○ Class B tertiary hospital
   - ○ Class A secondary hospitals
   - ○ Class B secondary hospitals
   - ○ Primary hospital
8. Your department?
   - ○ Emergency Department
   - ○ General Surgery
   - ○ General Medicine
   - ○ Other (please specify) _________
9. Your title level is?
   - ○ No job title
   - ○ Primary title
   - ○ Middle title
   - ○ Vice-senior title
   - ○ Senior title
10. Have you received training in the diagnosis and treatment of snakebites？
    - ○ Yes
    - ○ No
11. Does the hospital where you work have antivenoms？
    - ○ Yes
    - ○ No

- Not clear

12. Do you know the way to obtain antivenoms？
    - Yes
    - No
13. Do you have any experience treating snakebite patients？
    - Yes
    - No
14. Your self-evaluation of the current snakebite treatment ability is
    - Very skilled
    - Proficient
    - Moderately skilled
    - Unskilled
    - Very unskilled

Diagnosis and treatment of snakebites(Multiple choice Please select all correct answers)

1. Venomous snake classification (Multiple choice question)

- Neurotoxicen venoming
- Cytotoxic venoming
- Haemotoxic venoming
- Mixed venoming
- Not know

2. What are the common Neurotoxicen venoming snakes? (Multiple choice question)

- Krait
- Five-footed Snake
- Pallas pit viper
- Sea snake
- Silver-banded krait
- Not know

3. When will systemic envenoming symptoms occur after being bitten by a neurotoxic snake? (Single choice question)
○ 1 (Hour (s))
○ 2 (Hour (s))
○ 3 (Hour (s))
○ Not know
4. What are the common Haemotoxic venoming snakes? (Multiple choice question)
○ Russell′ s viper
○ Pallas pit viper
○ Bamboo leaf green
○ Elapid snake
○ Soldering iron
○ Not know
5. What are the common cytotoxic venomous snakes? (Single choice question)
○ Elapid snake
○ Pallas pit viper
○ Russell′ s viper
○ Bamboo leaf green
○ Five-footed Snake
○ Not know
6. What are the common mixed venomous snakes? (Multiple choice question)
○ King cobra
○ Pallas pit viper
○ Agkistrodon halys
○ Five-footed Snake
○ Dinodon rufozonatum snake
○ Not know
7. What are the general symptoms of haemotoxic venoming snakebite? (Multiple choice question)
○ DIC-like reaction

- Nose bleeding, gum bleeding
- Hematuria
- Gastrointestinal bleeding
- Cerebral hemorrhage
- Respiratory dyspnea
- Blood coagulation loss
- Not know

8. What are the general symptoms after neurotoxicen venoming snakebite? (Multiple choice question)

- Eyelid ptosis, diplopia
- Dysphagia
- Respiratory paralysis, respiratory failure
- Myasthenia of limbs
- Swelling
- Hematuria
- Acute kidney injury
- Not know

9. What are the general symptoms of Cytotoxic venoming snakebite? (Multiple choice question)

- Soft tissue necrosis
- Soft tissue swelling
- Myocardial cell damage
- The wound is red, swollen and painful
- Multiple organ failure
- Systemic inflammatory response syndrome
- Rhabdomyolysis
- Not know

10. Snakebite wound shape? (Multiple choice question)

- . .
- ::

- ○ Two rows of neat and consistent depth of tooth marks
- ○ There are 1 ~ 4 teeth marks, 2 in general. The teeth marks are deep and thick, and the spacing is large
- ○ It is arranged in the shape of ' 八 ' or inverted ' 八 '.
- ○ Serrated or curved arrangement
- ○ Not know

11. The principle of treatment of venomous snakebites (Multiple choice question)

- ○ Quickly identify whether it is a venomous snakebites
- ○ Classification treatment
- ○ Remove the local venom immediately to prevent further absorption of the toxin
- ○ Antagonizing or neutralizing absorbed toxins
- ○ Use antivenoms as soon as possible according to the type of snake venom
- ○ Prevention and treatment of various complications
- ○ Strengthen symptomatic and supportive treatment
- ○ Not know

12. Indications for use of antivenom (Multiple choice question)

- ○ A definite or suspected diagnosis of a venomous snakebite accompanied by at least one manifestation of venoming
- ○ Symptoms of intoxication, local swelling over half of the bitten limb within 48 hours of the bite
- ○ Rapid progression of swelling
- ○ Symptom of systemic venoming, such as coagulation disorders, thrombocytopenia, systemic organs, organs of spontaneous bleeding, etc
- ○ Toxin reflux after bite leads to lymph node swelling and pain
- ○ There is no absolute contraindication of antivenoms for snakebite victims
- ○ Not know
