# Supplementary material for: Current Status and Influencing Factors of Snakebite Diagnosis and Treatment Knowledge Among Medical Staff in China: A Cross-Sectional Study
Source: Int J Public Health. 2023 Dec 11;68:1606601. doi: 10.3389/ijph.2023.1606601 (PMC10749458; doi:10.3389/ijph.2023.1606601)
Supplement: Supplementary file 5 [file Table2.DOC]

| **Supplementary Table2. Multicollinearity test results** | | |
| --- | --- | --- |
| **Variable** | **Tolerance** | **Variance**  **inflation factor** |
|
| Work tenure (years) | 0.67 | 1.50 |
| Region | 0.88 | 1.14 |
| Gender | 0.73 | 1.37 |
| Occupation | 0.66 | 1.53 |
| Education level | 0.72 | 1.38 |
| Level of hospital | 0.63 | 1.59 |
| Work department | 0.68 | 1.48 |
| Professional title | 0.60 | 1.68 |
| Training in the diagnosis and treatment of snakebites | 0.74 | 1.36 |
| Experiences in treating patients with snakebites | 0.60 | 1.67 |
| The hospital where they work has antivenom | 0.74 | 1.36 |
| Evaluation of their current ability to treat snakebites | 0.60 | 1.67 |
